# Behavior of technetium in nuclear waste vitrification processes

**DOI:** 10.1007/s10967-014-3900-9

**Published:** 2015-01-14

**Authors:** Ian L. Pegg

**Affiliations:** Vitreous State Laboratory, The Catholic University of America, 620 Michigan Avenue, NE, Washington, DC 20064 USA

**Keywords:** Technetium, Vitrification, Hanford, Technetium volatility, Technetium glass structure

## Abstract

Nearly 100 tests were performed with prototypical melters and off-gas system components to investigate the extents to which technetium is incorporated into the glass melt, partitioned to the off-gas stream, and captured by the off-gas treatment system components during waste vitrification. The tests employed several simulants, spiked with ^99m^Tc and Re (a potential surrogate), of the low activity waste separated from nuclear wastes in storage in the Hanford tanks, which is planned for immobilization in borosilicate glass. Single-pass technetium retention averaged about 35 % and increased significantly with recycle of the off-gas treatment fluids. The fraction escaping the recycle loop was very small.

## Introduction

Technetium is a fission product that is present in used nuclear fuel and wastes generated from nuclear fuel reprocessing. At the Hanford site in Washington State, approximately 24,000 Ci of ^99^Tc in about 56 million gallons of high-level waste (HLW) from the production of plutonium for nuclear weapons is currently stored in aging underground tanks. This waste will be separated into low-activity waste (LAW) and HLW fractions and separately converted to glass by vitrification in the joule-heated ceramic melters (JHCMs) in the Hanford Tank Waste Treatment and Immobilization Plant (WTP), which is under construction. The HLW glass is designed for disposal in a national HLW repository while the LAW glass will be disposed on site. The long half-life of ^99^Tc (211,000 years), coupled with the high environmental mobility of the very soluble pertechnetate anion, makes ^99^Tc one of the most significant risk contributors in performance assessments of the LAW disposal facility. The mode of incorporation of technetium into the glass structure as well as its volatility in the high-temperature vitrification process and subsequent capture in the downstream off-gas treatment systems are important to the overall performance of the treatment and immobilization process. Technetium is one of the more volatile radionuclides and its retention in LAW glass can vary depending on feed composition, feed chemistry, and melter operating parameters. High retention of technetium in the glass is desirable in order to minimize the fraction that is directed to secondary waste treatment and disposal in less durable non-glass waste forms.

The Hanford tanks contain, in varying amounts, HLW sludge formed by precipitation of most of the heavy metals and long-lived transuranics after neutralization of the acid wastes with sodium hydroxide, a residual high-sodium salt solution called supernate, and crystallized supernate called saltcake. The major radionuclides in the supernate are those that are soluble at high pH, which includes cesium and technetium. The WTP pretreatment facility is designed to separate the HLW solids from the supernate by cross-flow filtration and remove cesium from the supernate by ion exchange to produce the feed to the LAW vitrification facility. Liquid effluent streams from the LAW and HLW off-gas treatment systems are returned to the pretreatment facility, evaporated, and recycled to the melter feed. In principle, such recycle can achieve very high incorporation of even very volatile species into the glass product, depending on the effectiveness of the capture and recycle in the off-gas treatment systems. This paper describes testing that was performed to investigate the retention of technetium in the LAW glass with and without recycle and its capture efficiency in the off-gas treatment system. Since rhenium is often used as a non-radioactive surrogate for technetium, direct comparisons between the behavior of these species were also made.

Technetium and rhenium chemistry tends to be dominated by the most stable VII oxidation state and the next most stable IV state. In general, technetium and rhenium species with oxidation states of less than IV are rapidly oxidized and those with oxidation states between IV and VII tend to disproportionate into corresponding mixtures of IV and VII compounds [1]. For both elements, the VII oxides are more volatile than the IV oxides [1]; their stability fields have been mapped as a function of oxygen fugacity [2]. Tc_2_O_7_ boils at 311 °C, Re_2_O_7_ boils at 363 °C, and TcO_2_ sublimes at 900 °C [1]. Electrochemical measurements made in borosilicate glass melts have shown that technetium is more easily reduced than is rhenium [3], as also is the case in aqueous solutions. Similarities as well as important differences in the behavior of technetium and rhenium during vitrification of LAW simulants and subsequent vapor phase hydration testing of the resulting LAW glasses have also been reported [4–6]. In aggregate, however, for these and many other reasons, rhenium, while imperfect, remains the best known chemical surrogate for technetium [1].

The retention of technetium and rhenium during the formation of borosilicate glass melts has been reviewed previously [1]. A general finding is that retention is increased under more reducing conditions, which favor the IV oxidation state over the VII state. For example, in crucible melt studies, Freud et al. [3] found technetium retentions of 45 and 75 % under oxidizing and reducing conditions, respectively, while Vida [7] found technetium retentions of between 47 and 70 % under reducing conditions. Darab and Smith [1] found similar retentions (~65 %) for rhenium in crucible melts with Hanford LAW simulants. Nine crucible melts made with samples of actual LAW showed technetium retentions of 12–63 % (one was 99 %), with an average of 38 % [8]. Small-scale JHCM tests using LAW simulants that were spiked with ^99m^Tc showed that 18–77 % of the technetium was retained over a wide range of process conditions [9]. Technetium retentions of about 38 % were observed during HLW treatment in the PAMELA JHCM in Mol, Belgium [1]. Overall, technetium retention in glass has been observed to vary widely depending on a number of factors including feed chemistry, redox, and process conditions such as melt temperature, cold-cap coverage, melt pool bubbling, etc.

It is important to note that incorporation of technetium into the glass melt is limited by volatility and not solubility. Homogeneous LAW glasses with over 1,500 ppm Tc have been made and characterized [4–6] and the solubility has been estimated at around 2,500 ppm [10], whereas the average concentration in the WTP LAW glass is expected to be about 3 ppm. Studies using X-ray absorption [4, 5, 10] and Raman spectroscopy [6, 11] have shown that technetium is present in these glasses as both Tc(VII) and Tc(IV), with the former dominating under the redox conditions expected for LAW vitrification. Conversely, while Re(VII) is observed, Re(IV) is not [5].

In JHCMs, waste and glass forming chemicals or glass frit are fed as an aqueous slurry onto the surface of the molten glass pool to form a “cold cap,” where a number of melt-rate controlling physical and chemical reactions occur. As the feed materials travel downward through the cold cap, water is evaporated, salts are melted and decomposed, and the products combine to form molten glass that then becomes part of the underlying melt pool. It is likely that technetium species are first incorporated into low-melting salt phases, which, for LAW feeds are primarily nitrates. The WTP LAW flow-sheet includes sugar additions to counter-balance the oxidizing effects of nitrates in the LAW feed in order to mitigate melt pool foaming. Sugar and other reductants can also be used to promote the formation of more reducing conditions in the cold cap in order to favor the IV oxidation state and thereby increase technetium retention. However, to be viable, such an approach must also avoid the creation of overly reducing conditions that can lead to the formation of deleterious phases, such as molten metals and sulfides that can compromise melter life. Such approaches were also investigated in the present work.

## Experimental

Testing was performed on a continuously-fed DM10 JHCM system that produced glass at a rate of about 50 kg per day [12–15]. The energy required to melt the feed is dissipated by resistance heating by passing an electric current between Inconel 690 plate electrodes that are submerged in the molten glass pool on opposite walls. An air-bubbler was used to stir the melt and increase the glass production rate. For the single-pass retention tests (i.e., without recycle), the melter was fitted with a dry off-gas treatment system employing filtration stages only. For the recycle tests, the melter was fitted with an off-gas treatment system that included a submerged bed scrubber (SBS), wet electrostatic precipitator (WESP), and HEPA filtration, which is representative of the primary components used in the WTP. The liquid effluents from the SBS and WESP were concentrated in a vacuum evaporator in real time and the concentrate was recycled back to the melter feed. The tests employed seven simulated LAW streams representing pretreated supernate from Hanford tanks AZ-101, AZ-102, AP-101, AN-102, AN-104, AN-105, and AN-107 and associated glass formulations (denoted LAWE3 through LAWE10H). These streams are essentially high-sodium salt solutions containing many components but predominantly nitrate, nitrite, hydroxide, aluminum, phosphate, potassium, sulfate, and chloride as well as various organics. The melter feed material was an aqueous slurry of the simulated waste mixed with glass forming chemicals (which provide sources of Si, B, Al, Fe Ca, Mg, Ti, Zn, Zr, Li), which is pumped continuously onto the surface of the molten glass pool in the melter. The glass is poured periodically from the melter using a prototypical air-lift discharge system.

The short-lived isotope of technetium, ^99m^Tc (half-life = 6 h) in the pertechnetate form, was used in place of ^99^Tc (half-life = 210,000 years). ^99m^Tc has easily detectable gamma emissions around 140 keV permitting analysis by gamma counting, which is fast and accurate. The short half-life means that the test systems and associated wastes are essentially decontaminated simply by allowing time for decay. However, the samples have to be collected and analyzed quickly, which, for a complex system that generates many samples, presents logistical challenges that have to be overcome. All measured activities were corrected to a common time.

Scale-up tests with technetium and rhenium were performed on a DM100 JHCM system, which is over five times larger than the DM10 system, and with rhenium on a DM1200 JHCM system, which is 60 times larger than the DM10 system [12, 13].

The melter feeds were spiked with ^99m^Tc at typically 1 mCi per kg of glass if all were retained; this corresponds to a technetium concentration in glass of about 0.2 ppt. Similarly, Re (in the perrhenate form) was added at typical concentrations of 400–900 ppm. The results from tests that were conducted under various conditions with and without rhenium showed that there was no discernable effect of the presence of rhenium on technetium retention [12].

## Results and discussion

### Single-pass retention

Eighty-five DM10 melter tests totaling about 1,100 h of testing, two DM100 melter tests totaling about 100 h of testing, and one DM1200 test producing six metric tons of glass were conducted [12, 13]. For each test, a mass balance for all feed constituents was performed across the feed, glass pool, discharge glasses, melter exhaust, exhaust from the primary off-gas system components, and the off-gas system sump solutions. Average mass balance closures were 97 % for technetium and 102 % for rhenium.

**Fig. 1:**
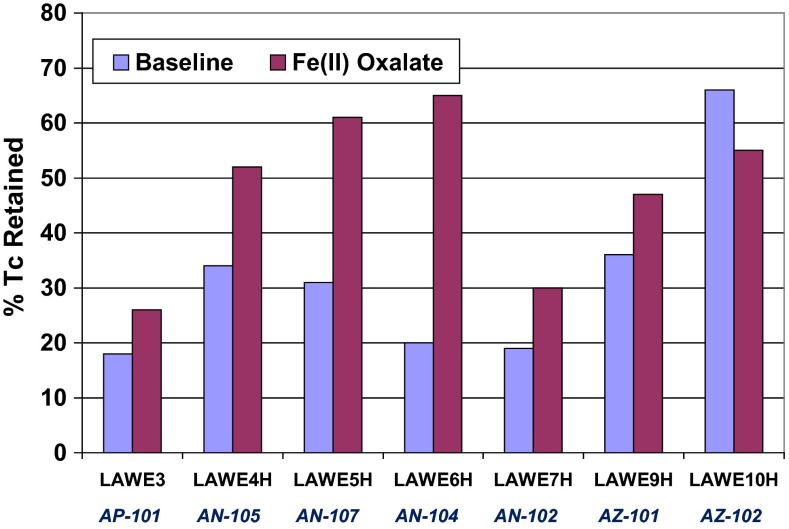
Measured single-pass technetium retentions for seven waste simulants and corresponding glass formulations with and without ferrous oxalate

As shown in Fig. 1, the amount of technetium and rhenium retained in the glass product varies widely across the seven LAW streams investigated and averaged about 35 %. The primary factor underlying the observed variation of retentions across waste types appears to be the nitrate content, with a lesser effect from the nitrite content. Retentions decrease as these species increase in the melter feed. As further corroboration of this effect, the retentions of technetium and rhenium in the glass product decrease approximately linearly with increasing nitrogen oxide emissions. Technetium retention improved as the conditions were made more reducing with the addition of organic additives such as sugar. However, none of the many organic reductants evaluated performed significantly better than sugar in terms of increasing retention without overly reducing the glass melt. Of the various methods investigated, the most effective method for enhancement of technetium and rhenium retention without excessive reduction of the glass melt was the use of iron(II) oxalate as an additive. Single-pass technetium retentions of up to 65 % were demonstrated using this method (Fig. 1). The primary mode of action of the iron(II) oxalate addition appears to be via reaction in the basic melter feed during which the divalent iron is oxidized by nitrate, destroying nitrate and thereby reducing its concentration in the melter feed. As noted above, the decreased nitrate content in the melter feed results in increased retention, presumably by decreasing the tendency to form the more volatile higher oxidation states of the species of concern. Iron(II) oxalate would therefore not be expected to provide an effective enhancement for wastes with very low concentrations of nitrates, as was observed experimentally in this work (AZ-101 and AZ-102 in Fig. 1).

**Fig. 2:**
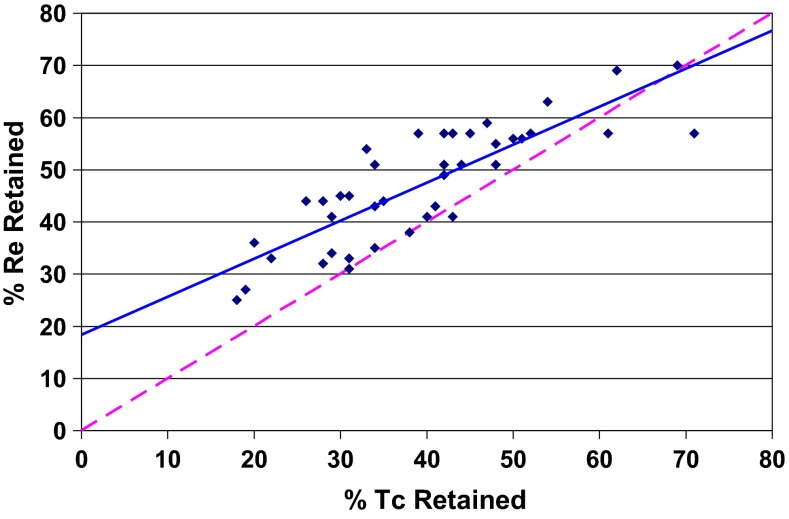
Comparison of single-pass technetium and rhenium retentions for all tests without ferrous oxalate

Overall, rhenium was shown to be a reasonable surrogate for technetium (Fig. 2) although on average rhenium retention in the glass product was higher than technetium in tests without iron(II) oxalate; the difference was greater at low retentions and near zero at high retentions, averaging ~7 absolute %). Tests with iron(II) oxalate showed a much poorer correlation between technetium and rhenium retentions and a distinct shift towards higher technetium retentions.

Tests to examine scale up from the DM10 to the DM100 (~5×) for melter feed with iron(II) oxalate as an additive showed remarkable consistency in the retentions of technetium, rhenium, and iodine across melter scales [12, 13]. Tests to examine scale up from the DM10 to the DM100 to the DM1200 (~60×) for melter feed with iron(II) oxalate as an additive showed remarkable consistency in the retentions of rhenium across melter scales [13].

Use of more reducing bubbling gases than air did not give any significant improvement in technetium retention during feeding (though there was improvement during idling). Processing at a lower glass pool temperature resulted in modestly increased technetium retention. However, such a mode of operation has the disadvantage that the glass production rate decreases significantly with decreasing temperature, which is economically undesirable.

**Fig. 3:**
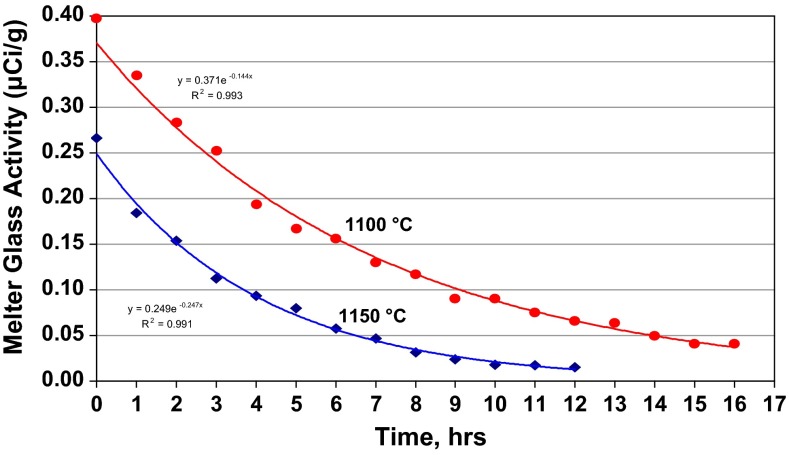
Loss of technetium from the melt pool during idling at two temperatures

Technetium was lost rapidly from the glass pool during idling (i.e. at nominal conditions but without feeding and hence without a cold cap). As shown in Fig. 3, the loss follows first-order kinetics, as would be expected, with a rate constant of about 0.25 h^−1^, which decreases with decreasing temperature, lower bubbler gas flow rate, and the use of more reducing bubbler gases [12].

### Retention with recycle

In the WTP system, technetium can exit the recycle loop via two routes: In the off-gas stream exiting the WESP and in the liquid condensate from the vacuum evaporator. Constituents in the off-gas stream from the WESP are further removed in a packed bed scrubber (PBS). The PBS effluent and evaporator condensate secondary waste from the WTP are directed ultimately to non-glass waste forms. Consequently, determination of the fraction of technetium exiting the recycle loop via these routes is important. There have been no previous measurements of this fraction. All seven LAW waste compositions were processed in nine nominally 72-h tests on the DM10 JHCM system with recycle, which permit such estimates to be made [14, 15].

With recycle, retentions of technetium and rhenium in the glass product were increased by factors of at least 2–3 over the corresponding single-pass values for almost all glass compositions. The average technetium and rhenium retentions in glass across all compositions tested were 74 and 79 %, respectively. All but two compositions showed technetium retentions in glass of 74–90 % and rhenium retentions in glass of 70–100 %. The increase in glass retention was limited by holdup of material in the system, particularly in the WESP internals, the film cooler, and the transition line. Mobilization of this material in order to make it available for recycle would likely further increase the retention in glass. The fraction of feed technetium exiting the recycle loop through the evaporator overheads was less than 0.03 % during normal operations and much lower for many tests. The fraction of feed technetium exiting the recycle loop through the WESP exhaust ranged from 0.01 to 0.5 % during normal operations. However, the fraction of feed technetium exiting the recycle loop through the WESP exhaust was critically dependent on the performance of the WESP and increased to above 10 % (i.e., by a factor of about 500 or more) during periods of WESP malfunction, highlighting the high removal efficiency of the WESP.

For technetium, the system component retention factors averaged across all seven feed types were: single-pass melter: 35.2 % (vs. 74 % average with recycle); SBS: 52.2 %; WESP: 99.8 %. For rhenium, the system component retention factors averaged across all seven feed types were: single-pass melter: 43.1 % (vs. 79 % average with recycle); SBS: 70.0 %; WESP: 99.4 %. The single-pass melter retentions for technetium and rhenium measured in the recycle tests show good agreement with the corresponding values measured in the single-pass tests on the DM10 and DM100 melter systems. The SBS and WESP retention factors measured for rhenium show excellent agreement with those measured on the DM1200 system, which is 60 times larger.

**Fig. 4:**
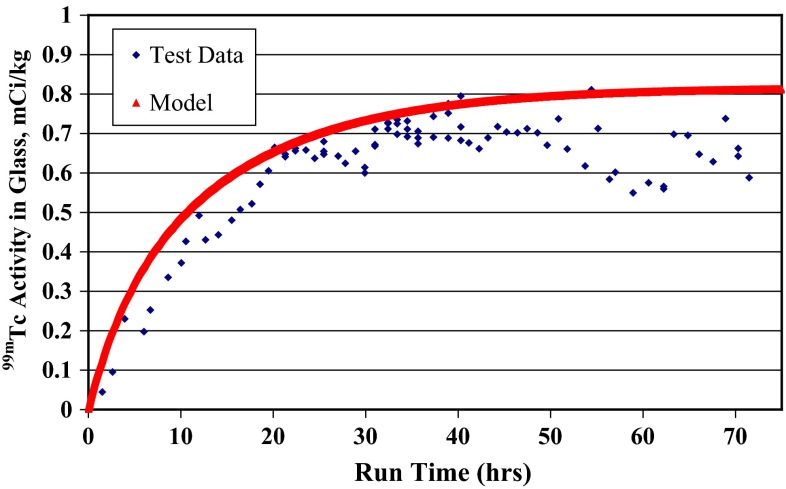
Comparison of measured technetium concentration in the glass product with that calculated from a system process model with no adjustable parameters

Overall, technetium and rhenium showed remarkably similar distribution across the various system sumps. However, as noted above, the retention factor for technetium in the SBS was significantly lower than that for rhenium, both on average and in all but one of the individual tests. The steady state retention of technetium in the glass product showed a reasonable correlation to that for rhenium, with the average rhenium retention being roughly 10 % absolute higher than that for technetium, similar to what was observed in the single-pass tests. The technetium mass balance closure reached as high as 99 % but averaged about 94 %, which is about 8 % lower than that observed for rhenium. When the measured retention factors for technetium and rhenium for each of the system components are input into a process model that was developed for the system, reasonable agreement with measured glass data was found, as shown in Fig. 4 [14, 15].

The amount of technetium and rhenium retained in the glass product showed similar variations across the seven LAW streams investigated to that observed in the tests without recycle but the variation was somewhat less pronounced. Testing with a high potassium feed showed higher emissions of both technetium and potassium in the WESP exhaust, suggesting that the presence of potassium may reduce the capture efficiency of technetium in the off-gas system. Technetium retention in the glass product was more sensitive to interruptions in the feed and recycle streams than was the case for rhenium suggesting that data from tests with technetium, rather than a rhenium surrogate, should be used in evaluating the potential impacts of such interruptions on the performance of the WTP.

## Conclusions

The mean single-pass technetium retention in the glass melt over seven different waste compositions was about 35 % but reached as high as 65 % with the addition of ferrous oxalate to the melter feed. Recycle increased the technetium retention significantly, in accord with process models. Thus, at steady state, it should be possible to achieve near complete incorporation of technetium into the glass product. Hold up of technetium in the system piping and at other locations where it is not re-mobilized and made available for recycle could limit this, however. Highly effective capture in the off-gas system (52.2 % in the SBS and 99.8 % in the WESP) made the fraction of technetium escaping the recycle loop very small. The fraction of feed technetium exiting the recycle loop through the evaporator overheads was less than 0.03 % during normal operations and much lower for many tests. The fraction of feed technetium exiting the recycle loop through the WESP exhaust ranged from 0.01 to 0.5 % during normal operations but increased to above 10 % during periods of WESP malfunction. In general, rhenium behavior tracked that of technetium reasonably well but exceptions were evident.
